# Estimating the In Vivo Killing Efficacy of Cytotoxic T Lymphocytes across Different Peptide-MHC Complex Densities

**DOI:** 10.1371/journal.pcbi.1004178

**Published:** 2015-05-01

**Authors:** Victor Garcia, Kirsten Richter, Frederik Graw, Annette Oxenius, Roland R. Regoes

**Affiliations:** 1 Institute of Integrative Biology, ETH Zurich, Universitätstr, Zurich, Switzerland; 2 Institute of Microbiology, ETH Zurich, Zurich, Switzerland; 3 Center for Modeling and Simulation in the Biosciences, Bio-Quant Center, Heidelberg University, Heidelberg, Germany; La Jolla Institute for Allergy and Immunology, UNITED STATES

## Abstract

Cytotoxic T lymphocytes (CTLs) are important agents in the control of intracellular pathogens, which specifically recognize and kill infected cells. Recently developed experimental methods allow the estimation of the CTL's efficacy in detecting and clearing infected host cells. One method, the in vivo killing assay, utilizes the adoptive transfer of antigen displaying target cells into the bloodstream of mice. Surprisingly, killing efficacies measured by this method are often much higher than estimates obtained by other methods based on, for instance, the dynamics of escape mutations. In this study, we investigated what fraction of this variation can be explained by differences in peptide loads employed in in vivo killing assays. We addressed this question in mice immunized with lymphocytic choriomeningitis virus (LCMV). We conducted in vivo killing assays varying the loads of the immunodominant epitope GP33 on target cells. Using a mathematical model, we determined the efficacy of effector and memory CTL, as well as CTL in chronically infected mice. We found that the killing efficacy is substantially reduced at lower peptide loads. For physiological peptide loads, our analysis predicts more than a factor 10 lower CTL efficacies than at maximum peptide loads. Assuming that the efficacy scales linearly with the frequency of CTL, a clear hierarchy emerges among the groups across all peptide antigen concentrations. The group of mice with chronic LCMV infections shows a consistently higher killing efficacy per CTL than the acutely infected mouse group, which in turn has a consistently larger efficacy than the memory mouse group. We conclude that CTL killing efficacy dependence on surface epitope frequencies can only partially explain the variation in in vivo killing efficacy estimates across experimental methods and viral systems, which vary about four orders of magnitude. In contrast, peptide load differences can explain at most two orders of magnitude.

## Author Summary

The immune system reacts to the presence of a viral pathogen within the host by the elicitation of an immune response. This response is characterized by the activation and proliferation of specific cell types, which, for instance, produce neutralizing antibodies or kill cells infected by the virus. Cytotoxic T lymphocytes (CTLs) function as an important protecting element of the system by recognizing and clearing infected viral target cells. Surprisingly, estimates of the killing efficacy of CTLs vary about four orders of magnitude across experimental methods and viral systems. In some studies, CTL killing efficacies were estimated by employing pre-treated cells that mimick virus infected cells. In general, cells signal their infection by a pathogen to the immune system by presenting viral peptides on their cellular surface. For the experimentally pretreated cells, these peptides were artificially loaded onto the surface at very high densities. In this paper, we study to what extent the variation in peptide densities can explain the variation found in killing efficacy estimates across methods and viral systems. We found that peptide densities explain only up to two orders of magnitude in killing efficacy variation. The remaining variation must originate from other sources, which might be specific to the viral study system.

## Introduction

Adaptive immune responses exert important selective pressures on viral infections through various mechanisms, such as neutralization of virus particles by antibodies or killing virus-infected cells by cytotoxic T lymphocytes (CTLs). Efforts to quantify the ability of CTLs to kill infected host cells have yielded results with considerable variation [1, 2]. In fact, estimates of the efficacy of CTLs at recognizing and clearing infected viral target cells *in vivo* vary by several orders of magnitude between experimental designs and viral study systems [1, 3, 3–16].

*In vivo* CTL killing efficacy estimates exist for the following types of viral study systems: HIV/SIV [4–11], lymphocytic choriomeningitis virus (LCMV) [3, 12–15], polyoma virus [16], HTLV-1 [1], and bovine leukemia virus (BLV) [1]. The killing efficacy of CTLs in HIV [5, 6], SIV [4, 9, 10], HTLV-1 [1], and bovine leukemia virus infection [1] yield distinct, relatively low estimates. These estimates capture the rate at which a target cell is cleared by the total CTL response, and range from 0.1d^−1^ to 10d^−1^ [1]. In contrast, polyoma virus and LCMV have been shown to yield high killing efficacy estimates of 20−500d^−1^ for epitope-specific clones in either acute or chronic infections [1, 3, 13–17]. Hence, compared to LCMV and polyoma virus, HTLV-1 and BLV yield much lower estimates.

The variation in these estimates might be primarily due to the viral study systems. The experimental methods employed to obtain the estimates for HIV/SIV, HTLV-1 and BLV rely on distinct approaches. HIV estimates are based on two approaches: the injection of CTL [5] and on the observation of viral escape mutants [4, 6], with an almost hundredfold difference between some estimates [1]. In contrast, the HTLV-1 and BLV estimates are based on assessing the decay rate of labeled cells, in a similar fashion as employed for LCMV and polyoma virus [1].

Estimates for LCMV and polyoma virus rely on an experimental technique referred to as *in vivo* killing assay. This technique involves the injection of target cells, loaded with viral peptides on their surface, into the bloodstream of mice. The subsequent tracking of their disappearance in relation to unpulsed control cells allows for the estimation of the effect of killing by CTLs. In most of these assays, the number of viral peptides that each target cell displays is unnaturally high, three orders of magnitude higher than on naturally infected cells (see Discussion).

In part, the discrepancy between the *in vivo* CTL efficacy estimates across experimental methods and study systems is likely to arise from the unnaturally high peptide loads on target cells in *in vivo* killing assays. The highest per CTL killing efficacies have been measured in LCMV in vivo killing assays with high peptide loads [14]. When reducing peptide loads to very low levels which make target cells unrecognizable by CTL, we accordingly expect a decrease per CTL killing efficacies to almost zero. Hence, peptide load variation could theoretically explain the entire variation in CTL killing efficacies.

In this study, we investigate to what extent CTL killing efficacies depend on peptide load on target cells, and what proportion of the entire CTL efficacy variation can be explained by this effect. To this end, we conducted CTL *in vivo* killing assays in mice acutely or chronically infected with LCMV Docile. During chronic infections, CTLs are assumed to become dysfunctional or exhausted, displaying poor effector function [18]. We estimated the killing efficacies of effector, memory and chronic-infection CTLs for different peptide loads using mathematical models that extend previous approaches [15]. The types of CTL were classified by the administered dose of the LCMV Docile and the time after infection (see Materials and Methods).

We find that the per cell killing efficacy first increases with peptide loads, and saturates above a peptide load of approximately 10^−1^ *μg*/*ml*. For physiologically reasonable peptide loads however, which we estimate to lie around 10^−3^ *μg*/*ml* (see Discussion), the CTL efficacy is only one order of magnitude lower than at saturation for all three CTL types. This leaves most of the differences in *in vivo* CTL efficacy estimates unexplained.

We also found that individual CTL during chronic infections kill cells with physiological peptide loads at a higher rate than effector or memory CTL. This result needs to be interpreted in the context of whether exhausted CTLs display reduced killing efficacies [18]. As in studies with high peptide loads [15], we found no evidence that CTL killing in chronic infections is impaired.

## Materials and Methods

### Experiments

#### Ethical statement

Following the Felasa-recommmendation 2002 for laboratory animals, C57BL/6 mice were kept under specific pathogen-free conditions. Animal experiments were performed according to the guidelines of the animal experimentation law (SR 455.163; TVV) of the Swiss Federal Government. The protocol was approved by the Cantonal Veterinary Office (animal experimentation number 127/2011).

#### Virus and viral peptides

The employed LCMV Docile was obtained by propagation on MDCK cells at low multiplicity of infection. We employed methods described in [19] for the quantification of infectious virus titres. Viral peptides used for pulsing on target cells (GP33-41;KAVYNFATM) were purchased from NeoMPS (Strasbourg, France).

#### Cytotoxicity assay

**Fig 1:**
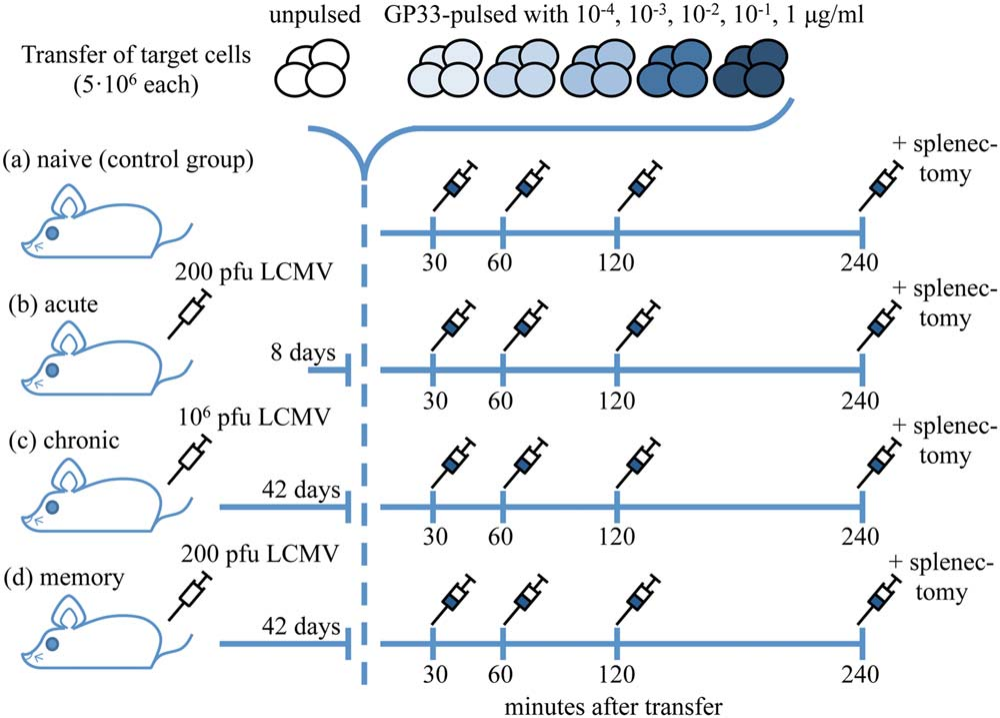
Experimental design of the *in vivo* killing assay performed on four treatment groups of four mice. Mice in the control group (a) were not challenged with LCMV, and stayed naïve. Mice in the acute (b) and memory (d) groups were infected with 200 pfu, while the chronic group (c) received 1 × 10^6^ pfu of LCMV Docile. After a delay of eight (acute group) and 42 days (chronic and memory groups) respectively, labelled target cells were injected intravenously into all mice including the naïve group. The transferred cells consisted of subpopulations of cells pulsed with different peptide loads, including a subpopulation of unpulsed cells. The subpopulations were pulsed with the indicated concentrations of GP33-epitope (5 × 10^6^ cells each). At the indicated time points, blood samples were taken and analysed for the proportions of the labelled cells by flow cytometry. Splenectomy was conducted on each mouse 4 hours after cell transfer and the splenocytes analysed for specific lysis of target cells and the proportions of epitope-specific CTLs. Note that in contrast to [15], this design uses an additional treatment group—the memory group, a larger number of target cells is transferred, and the time between infection and cell transfer is tuned to observe the desired effector, chronic and memory CTLs.

LCMV Docile is known to cause acute or chronic infections depending on the inoculum size of viral particles infecting the host [20]. We infected four groups of four female C57BL/6 mice expressing the congenic marker Ly5.2 intravenously with LCMV-Docile at distinct times before injecting target cells (Fig 1.). The first group (acute group) was infected with an inoculum of 200 plaque forming units (pfu) eight days prior to transfer. Two further groups (chronic and memory groups) were infected with 10^6^ and 200 pfu, respectively, 42 days prior to transfer. One group (naïve group) was used as negative control group. The terms used to denote the treatment groups were chosen to describe the most prevalent CTL type found in mice infected with a specific inoculum dose and at the time of the measurement. For each mouse, the transferred target cell pool was comprised of 1.5 × 10^7^ homozygous splenocytes (and therefore Ly5.1+ Ly5.2-) and Ly5.1 heterozygous splenocytes (and therefore Ly5.1/Ly5.2 double positive).

**Fig 2:**
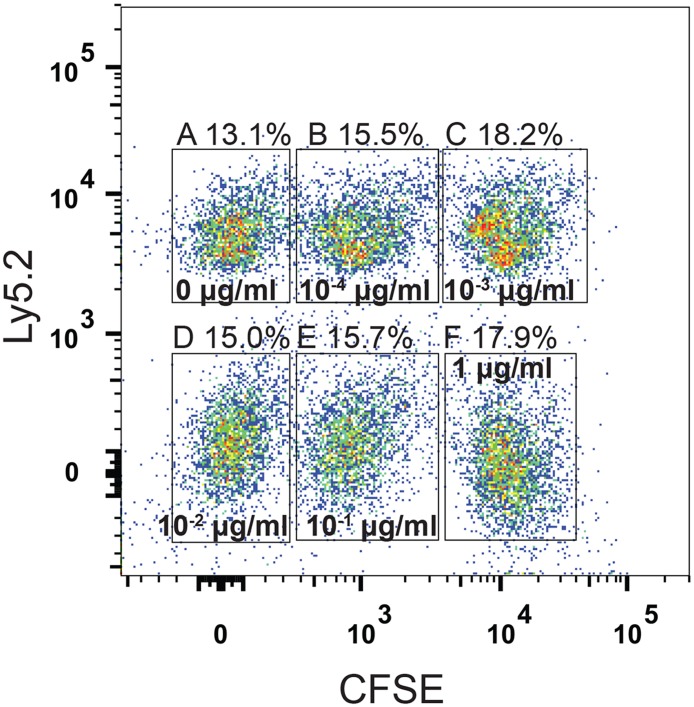
FACS analysis of target cells before transfer can reliably differentiate target cell subpopulations. To assess the reliability of our experimental design, we tested whether the six target cell subpopulations pulsed at different peptide concentrations could be distinguished by their markers alone. Before FACS analysis, equal numbers of the different target cell populations were mixed. The six target cell subpopulations –no peptide in A), and pulsed with increasing GP33 peptide concentrations of $10^{-4}\frac{\mu \text{g}}{\text{ml}}$ in B) to $1\frac{\mu \text{g}}{\text{ml}}$ in F)– employed in our study could reliably be differentiated according to their Ly5.1/Ly5.2 expression and CFSE staining intensity. The percentages of detected target cells are given above the panels A) to F).

With this design, it is possible to differentiate between cells originating from the recipient mice, and the cells from the two transferred subpools (see Fig 2.). 5 × 10^6^ cells in the first Ly5.1/Ly5.1 subpool were pulsed with $10^{0}\frac{\mu \text{g}}{\text{ml}}$ LCMV derived GP33 peptide for one hour at 37°C, and were stained with 2.5*μ*M CFSE. Similarly, two further 5 × 10^6^ cells of the same subpool were pulsed with $10^{-1}\frac{\mu \text{g}}{\text{ml}}$ GP33 (stained with 0.25*μ*M CFSE) and $10^{-2}\frac{\mu \text{g}}{\text{ml}}$ GP33 (not stained with CFSE), respectively. The Ly5.1/Ly5.2 subpool was stained analogously to the first (2.5*μ*M, 0.25*μ*M and no CFSE), but pulsed with concentrations $10^{-3}\frac{\mu \text{g}}{\text{ml}}$, $10^{-4}\frac{\mu \text{g}}{\text{ml}}$ and and no peptide (the unpulsed subpopulation), respectively.

This design allows us to distinguish between all six subpopulations pulsed with different peptide concentrations (including the unpulsed) by measuring the intensity of the CFSE dye as well as the presence or absence of Ly5.1 and Ly5.2 congenic markers. Blood was harvested at 30, 60, 120, 240 min after transfer and analyzed for Ly5.2 and Ly5.1 expression, as well as CFSE intensity.

Four hours after the transfer, the mice were sacrificed and splenectomy conducted in order to generate single cell suspensions. Splenocytes were analyzed for CD8 and GP33 tetramer staining, as well as CFSE intensity and Ly5.1, Ly5.2 expression. All the relevant data obtained from this experiment are given in S1 Data.

#### Flow cytometry

The antibodies for FACS were purchased from LucernaChem (Luzern, Switzerland). For the generation of peptide/MHCI tetramers we followed [21]. Multiparameter flow cytometric analysis was performed using a FACS LSRII flow cytometer (BD Biosciences, Allschwil, Switzerland) with FACSDiva software. Analysis was performed using FlowJo software (TreeStar, San Carlos, CA).

### Statistics

We adopt a statistical framework which allows us to estimate the killing efficacy dependence on the pulsing concentration of peptides on target cells separately for each of the treatment groups (acute, chronic and memory groups), and to obtain estimates for the combined data set of all groups.

Following the notation established in [15], we index mice with *i* = 1, …, *m*, and label the time points *t*_*l*_, where *l* = 1, ⋯, *L*. Additionally, we have target cells pulsed at increasing concentration *λ*_*d*_, where *d* = 1, …, *D*. For each mouse *i* the experimental methods allow us to retrieve the proportions of transferred target cells pulsed at concentrations *λ*_*d*_, ${\tilde{F}}_{\lambda_{\text{d}},i}(t_{l})$, as well as for unpulsed cells, ${\tilde{F}}_{\text{u},i}(t_{})$ in the blood at each time point. These data permit to estimate the probability of a target cell in mouse *i* pulsed with peptide concentration *λ*_*d*_ to be killed by the GP33-specific CTL response until time *t*_*l*_:

$$\begin{array}{c} {\hat{p}}_{i,\lambda_{d}}\left(t_{l}\right)=1-\frac{{\tilde{F}}_{\lambda_{d},i}\left(t_{l}\right)}{f_{d}{\tilde{F}}_{u,i}\left(t_{l}\right)}, \end{array}$$ (1)

where as in [15], *f*_*d*_ is used to correct for the different ratios that might arise in the inoculum. To estimate *f*_*d*_, we calculated the ratios of cells pulsed with concentration *λ*_*d*_ and unpulsed cells at each time point *t*_*l*_ in naïve mice, and took the average of those values over all time points. The key assumption for this procedure is that CTLs do not affect the proportions of transferred target cells in naïve mice.

To estimate CTL efficacies and other quantities of interest in the killing assay data, a mathematical model that predicts the fraction of killed target cells at time *t*_*l*_ and peptide concentrations *λ*_*d*_, *p*(*t*_*l*_; *λ*_*d*_) (see Results), is fitted to the data ${\hat{p}}_{i,\lambda_{d}}(t_{l})$. For such a mathematical model two assumptions need to be made for the killing. First, how peptide load *λ* affects the killing rate constant *k* of CTLs has to be specified. Here, we assume a model with two parameters *k*_max_ and $\lambda_{\frac{1}{2}}$: $k(k_{\text{max}},\lambda_{\frac{1}{2}},\lambda )$ (see Results for interpretation of these parameters).

Second, a further assumption is required as to how the total killing efficacy is affected by the presence of other CTLs within the same compartment. Here, a general model might describe the net effect of the overall epitope-specific CTL frequency *g*(*C*), where *C* denotes the proportion of CTLs specific to the pulsed epitope among all splenocytes. The total killing —usually denoted by *f*(*k*, *C*) [3]— is captured by a mathematical expression incorporating both killing dependence on pulsing load and CTL saturation: $f\left(k(k_{\text{max}},\lambda_{\frac{1}{2}},\lambda_{d}),g(C_{i})\right)$.

Fitting is realized by employing a least square algorithm on the arcsin-square-root transformed data and probabilities [22]. The expected killing probabilities depend upon the model of choice, as shown in (3). Hence, the algorithm minimizes:

$$\begin{array}{c} \sum_{d=1}^{D}\sum_{i\in I_{T}}\sum_{l=1}^{L}(\arcsin{\left(\sqrt{\hat{p}(t_{l};f(k\left(k_{\max},\lambda_{\frac{1}{2}},\lambda_{d}\right),g\left(C_{i}\right)))})-\arcsin(\sqrt{p_{i,\lambda_{d}}\left(t_{l}\right)})\right)}^{2}, \end{array}$$ (2)

where *I*_*T*_ denotes the index set of mice over which the minimization is carried out. These data can stem from the mice within the same treatment group, or from a combined data set from all treatment groups. The parameters in the killing model *f*(*k*, *C*), such as *k*_max_ and $\lambda_{\frac{1}{2}}$, are estimated by incorporating the model into Eq (3), and fitting it to the observed fraction of pulsed target cells (1).

We used the R language for statistical computing [23] for the mathematical analysis performed on the data.

## Results

### (a) CD8^+^ T cell responses

**Fig 3:**
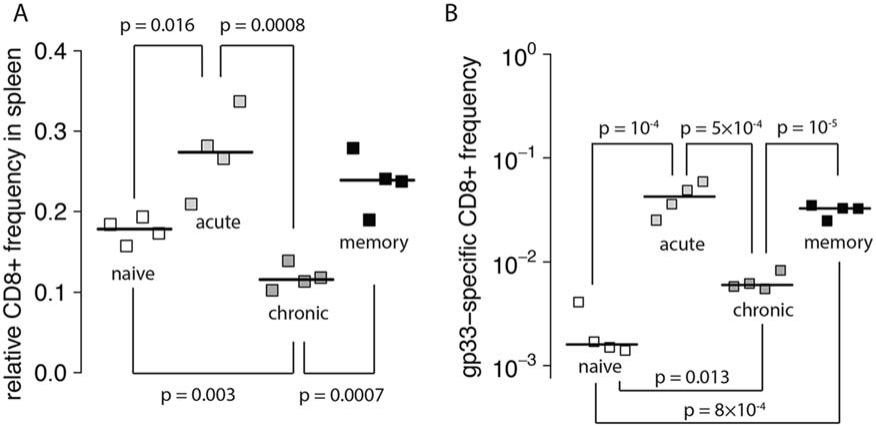
Frequency of CD8^+^ T cells in the spleen 4 hours after transfer of the target cells. Following the experimental design, splenectomies were conducted on the mice of all treatment groups. Panel A) shows the total frequencies of CD8^+^ T cells and B) the frequencies of the GP33-specific CD8^+^ T cells among splenocytes in naïve (unfilled), acute (light grey), chronic (dark grey) and memory (black) treatment groups found in the spleen 4 hours after the transfer of target cells. The black connecting lines show significant deviations in the CD8^+^ T cell frequencies between groups, and are given with the associated p-values.

The acute, chronic and memory groups differed in the overall frequencies of CD8^+^ T cells in the spleen four hours after the transfer of target cells (Fig 3A.). We performed two-tailed t-tests on the log-transformed frequencies among all groups to assess the inter-group differences. Applying the Holm-Bonferroni correction, we found that only half of the group pairs differed significantly. Acute and memory groups (*p* = 0.31), as well as the naïve and memory groups (*p* = 0.03) did not differ significantly in their total CD8^+^ T cell frequency. We also measured the GP33-specific CD8^+^ T cells in the spleen (Fig 3B.). All groups differed significantly, with the exception of acute and memory groups (*p* = 0.26). For both, the overall frequency of CD8^+^ T cells and the frequency of GP33-specific CD8^+^ T cells in the spleen, the acute and memory groups attained the highest values, whereas the number of cells in the chronically infected groups was lower compared to the acute and memory group but above the naïve group.

### (b) Target cell frequencies in blood and spleen

**Fig 4:**
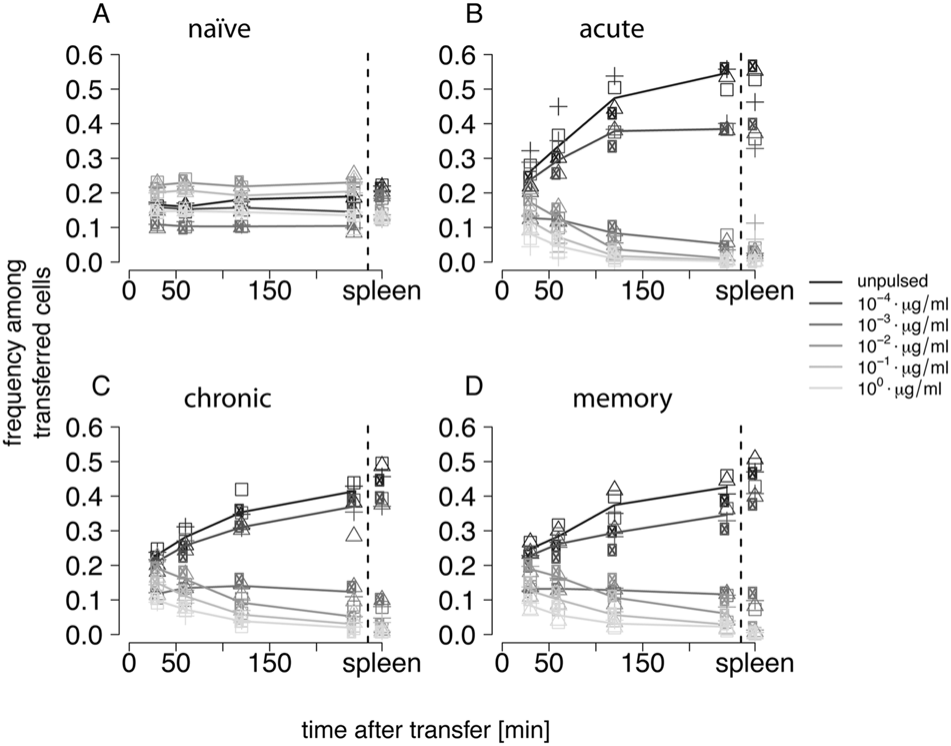
Relative frequency of target cells in the blood over time for the different mouse groups. The frequencies are given relative to the total target cell pool measured at 30, 60, 120 and 240 minutes after transfer. A) The frequencies of the target cells in the naive, B) acute, C) chronic and D) memory groups are shown for each individual mouse. The group means for each measurement are connected by lines specific to the pulsing concentration. The frequencies of the target cells found in the spleen after 4h are shown separately next to the dashed black line.

The level of pulsing of target cells heavily influenced their clearance by GP33-specific CD8^+^ T cells. The more peptides were loaded onto target cells before transfer, the faster the target cells disappeared relative to the total number of target cells. Fig 4. shows that in the naïve group relative target cell frequencies stayed approximately constant over the whole time course of the observation. In contrast, target cell frequencies for the other three groups show a significant reduction of most pulsed target cells when compared to unpulsed cells. Target cells which were pulsed at concentrations of $10^{-4}\frac{\mu g}{ml}$ of GP33 epitope increase in frequency in a similar fashion as the unpulsed cells, indicating that these pulsed cells are killed at very low rates.

### (c) Estimation of killing efficacy dependence on pulsing concentration

The measurement of the target cell frequencies relative to control cells contains information about how fast these target cells are cleared by CD8^+^ T cells. To extract this information, mathematical modeling is required to synthesize the migration of cells between the blood and secondary lymphatic tissues with the dynamics of killing by CTL. The mathematical model we used extends the previous model [15] by explicitly describing the dependence of the killing rate on the peptide load on the target cells.

To predict the proportions of killed target cells with a mathematical model, more detailed information is required as to how the target cells circulate in the blood and in which compartments they are killed. In previous work [15], we conducted experiments in naïve mice to determine at what rates target cells migrate to different compartments, such as the spleen, lung, liver and blood of mice. In these experiments, the total fraction of transferred target cells had been measured in all compartments over time. In a first step, we had observed that the target cells are likely to exclusively home to the spleen. For this reason, we assume that all target cells are killed in the spleen only. In a second step, we had calculated the net flux rate of target cells into the spleen, *μ*(*t*).

In the present study, we use the the estimate of *μ*(*t*) from [15]. The calculation of *μ*(*t*) is based on the existence of two compartments, a risk compartment –the spleen– where target cells can be cleared, and a non-risk compartment where they are not actively cleared. The net flux *μ*(*t*) between these two compartments constitutes a simplification to fluxes employed for inference in earlier models [3, 13] and should not be equated to such fluxes.

How efficiently the target cells are killed in the spleen by CD8^+^ T cells is determined by how abundant GP33-specific CD8^+^ T cells are in the spleen and the peptide load *λ*. These effects are mathematically captured in an expression for the total killing rate of the target cell population, *f*(*k*, *λ*, *C*). In this expression, *k* denotes the killing rate constant that describes the functional relationship between killing rate, CTL abundance *C*, and peptide load. The form of *f*(*k*, *λ*, *C*) will be specified below. We denote the proportion of killed target cells pulsed at concentration *λ* at time *t* by ${\hat{p}}_{i,\lambda }(t_{})$.

With the previously determined net flux rate *μ*(*t*) of cells between spleen and blood, and the total killing rate *f*(*k*, *λ*, *C*) we can derive the probability of a target cell to be killed by CTL’s until time t:

$$\begin{array}{c} p(t;k,\lambda ,C)=\\ \left\{ \begin{array}{cc} 1-(e^{\left(-\int_{0}^{t}\mu \left(s\right)\text{d}s\right)}+\int_{0}^{t}e^{\left(-\int_{0}^{u}\mu \left(s\right)\text{d}s\right)}\mu \left(u\right)e^{\left(-f(k,\lambda ,C)(t-u)\right)}\text{d}u) & \;\text{if}\;t\leq t_{0}\\ p\left(t_{0};k,\lambda ,C\right)+\int_{0}^{t_{0}}e^{\left(-\int_{0}^{u}\mu \left(s\right)\text{d}s\right)}\mu \left(u\right)e^{\left(-f\left(k,\lambda ,C\right)\left(t_{0}-u\right)\right)}\text{d}u\int_{t_{0}}^{t}e^{\left(\int_{t_{0}}^{u}\mu \left(s\right)-f\left(k,\lambda ,C\right)\text{d}s\right)}f\left(k,\lambda ,C\right)\text{d}u & \;\text{if}\;t>t_{0}, \end{array}\right. \end{array}$$ (3)

where, *t*_0_ denotes the time at which the netflux rate *μ*(*t*) becomes negative [15]. The derivation of this expression is based on the assumption that the target cells’ probability to be killed in the spleen depends on the target cells’ transition rate through the spleen, which roughly corresponds to *μ*(*t*). The dynamics of target cell circulation changes with the sign of *μ*(*t*): If *μ*(*t*) is positive, the probability of a target cell to be located in the spleen, and hence to be killed by CD8^+^ T cells, increases. If *μ*(*t*) is negative, this risk decreases. A detailed derivation of Eq (3) is provided in the supplementary information of [15].

### (c1) Killing efficacy dependence model under mass-action kinetics

To fit Eq (3) to the observed frequencies of pulsed cells, ${\hat{p}}_{i,\lambda }(t_{})$, we need to specify how the killing rate *f*(*k*, *λ*, *C*) depends on the level of GP33-specific CD8^+^ T cells in the spleen and the peptide load *λ*. Here, we assume this dependence to be given by:

$$\begin{array}{c} f\left(k,\lambda ,C\right)=k\left(k_{\max},\lambda_{\frac{1}{2}},\lambda \right)C=\frac{k_{\max}\lambda }{\lambda_{\frac{1}{2}}+\lambda }C, \end{array}$$ (4)

**Fig 5:**
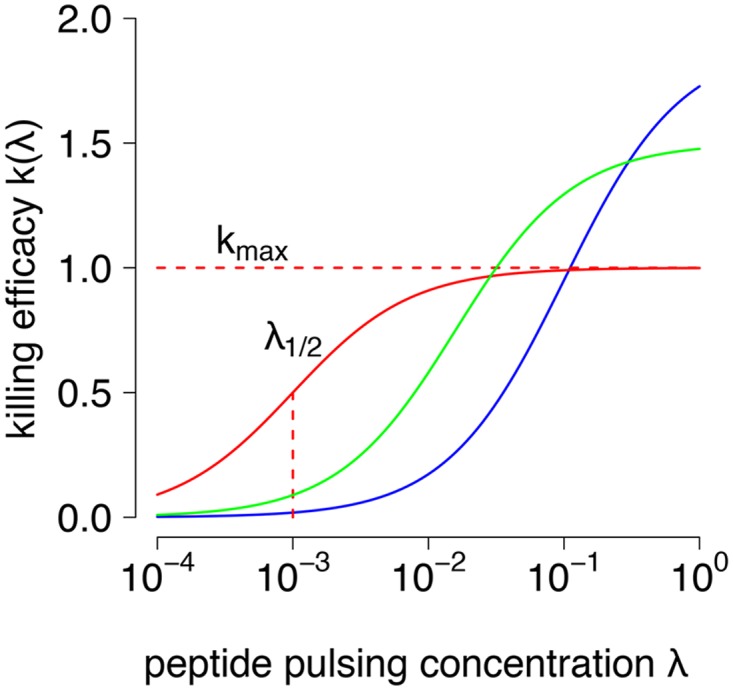
Curves of the killing efficacy model for different values of parameter pairs. The killing efficacy model $k(k_{\text{max}},\lambda_{\frac{1}{2}},\lambda )$ accounts for the varying efficacy of individual CD8^+^ T cells at killing target cells (*k*) at different peptide pulsing concentrations *λ*. The parameter *k*_max_ is the maximal killing rate of CD8^+^ T cells, where higher peptide concentrations *λ* do not increase CD8^+^ T cell killing. The parameter $\lambda_{\frac{1}{2}}$ describes at which peptide load killing is half-maximal. The lower this value, the more sensitive the CD8^+^ T cell is to peptide: a less sensitive CD8^+^ T cell would kill at lower rates at the same peptide concentrations. With this model, the comparison of two CTL types determined by distinct parameter pairs can lead to counter-intuitive results. For example, CD8^+^ T cells with low sensitivity but high maximal killing efficacy (blue line: *k*_max_ = 1.9, $\lambda_{\frac{1}{2}}=-1$) will be less efficient than more sensitive CD8^+^ T cells (red line: *k*_max_ = 1 and $\lambda_{\frac{1}{2}}=-3$ and green line: *k*_max_ = 1.5 and $\lambda_{\frac{1}{2}}=-1.8$) over a wide range of pulsed tetramer concentrations.

This expression assumes that the killing rate linearly increases with the frequency of GP33-specific CD8^+^ T cells, known as the mass-action killing assumption [24]. The parameters *k*_max_ and $\lambda_{\frac{1}{2}}$ characterize how the killing rate constant depends on the peptide load on the target cells (Fig 5.). *k*_max_ is the maximal killing efficacy and $\lambda_{\frac{1}{2}}$ accounts for the sensitivity of CTL’s to the frequency of presented epitopes on the target cell surface.

This model disentangles two aspects of the per cell killing efficacy *k*: the sensitivity parameter $\lambda_{\frac{1}{2}}$ determines the peptide loads required for a CTL to kill, while the parameter *k*_max_ describes the maximal killing capacity that can be reached per cell. In principle, it is possible that the hierarchy of killing abilities between CTL population depends on the peptide load on the target cells. For example, the CTL population described by the blue curve in Fig 5. is less sensitive to the peptide concentration on target cells than the other CTL populations and is therefore less efficacious, except for very high peptide loads.

We fitted function (3) assuming the killing efficacy dependence model defined in Eq (4) to the data. We found that the two parameters characterizing the killing rate in the mass-action killing model differed significantly between the acute, memory and chronic groups (F-test, *p* = 2 ⋅ 10^−33^). For this reason, we analyzed the three groups separately from this point onwards.

**Fig 6:**
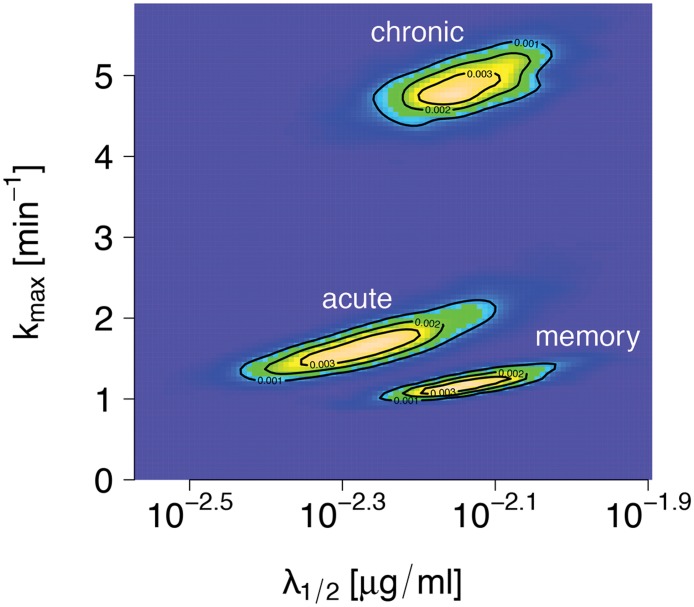
Likelihood landscapes from independent bootstrap procedures on the acute, chronic and memory treatment groups. Per group, 1000 bootstrap runs were repeated. A correlation between the estimates for *k*_max_ and $\lambda_{\frac{1}{2}}$ is visible for all three groups.

The maximum likelihood estimates for the maximum killing parameter *k*_max_ and the sensitivity $\lambda_{\frac{1}{2}}$, and their confidence intervals are listed in Table 1 and visualized in Fig 6. The estimates for *k*_max_ and $\lambda_{\frac{1}{2}}$ show a marked dominance of the maximum killing rate for chronically infected mice. The estimated maximal killing efficacy for the acutely infected mice (*k*_max_ = 1.71 min^−1^) is around three times smaller than the corresponding efficacy for chronically infected mice (*k*_max_ = 4.88 min^−1^), and about 1.4 times larger than the memory group mice (*k*_max_ = 1.19 min^−1^).

**Table 1:** Estimates of *k*_max_ and $\lambda_{\frac{1}{2}}$ for different treatment groups. The confidence intervals were calculated with 1000 bootstrap replicates and the percentile method.

**treatment group**	***k*_max_[min^−1^]**	**$\lambda_{\frac{1}{2}}[\frac{\mu \text{g}}{\text{ml}}]$**
(a) acute	1.71 (1.27, 2.41)	10^−2.24^ (10^−2.42^, 10^−2.05^)
(b) chronic	4.88 (4.33, 5.51)	10^−2.14^ (10^−2.28^, 10^−2.01^)
(c) memory	1.19 (0.96, 1.48)	10^−2.13^ (10^−2.26^, 10^−1.99^)

We calculated the associated minimal half-lifes by factoring in the proportion of GP33-specific splenocytes. The half-lifes calculated are 10.64 min, (95% CI: (6.65, 14.62)) for the acute, 22.58 min, (95% CI: (18.82, 26.33)) for the chronic and 19.09 min, (95% CI: (16.11, 22.08)) for the memory groups.

The decimal logarithm of the epitope recognition sensitivity was estimated to be highest for the acute group ($10^{-2.24}\frac{\mu \text{g}}{\text{ml}}$), and almost identical for the chronic and the memory groups ($10^{-2.14}\frac{\mu \text{g}}{\text{ml}}$, $10^{-2.13}\frac{\mu \text{g}}{\text{ml}}$ (Table 1)).

**Fig 7:**
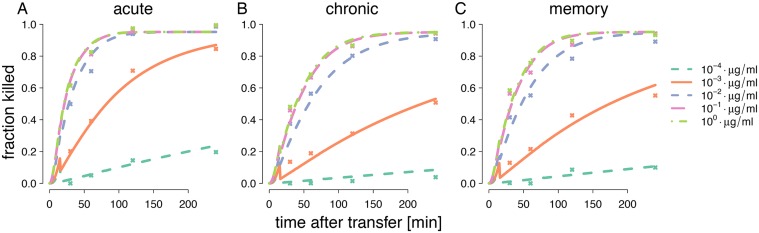
Fitted curves of Eq (3) assuming mass-action kinetics on estimated fractions of killed target cells in all mouse groups. The curves depend on the killing efficacy model $k(k_{\text{max}},\lambda_{\frac{1}{2}},\lambda )$ for distinct estimated group-specific values of parameter pairs (*k*_max_, $\lambda_{\frac{1}{2}}$) for A) the acute, B) the chronic and C) the memory groups. The dots are the averages of the fraction of killed target cells of all mice in a treatment group. The estimated values are given in Table 1. The five different lines for each group were calculated with Eq (3) using the group specific parameter pairs, as well as the pulsed peptide concentrations *λ*.

Fig 7. shows the fits of function (3) incorporating (4) for each group. The five curves shown for each group correspond to five peptide loads inserted into function (3), which is parametrized with the estimates in Table 1. Note that to generate all curves within a group only two parameter values are required. The simple model (4) is capable to successfully capture the most pronounced features of the data remarkably well.

**Fig 8:**
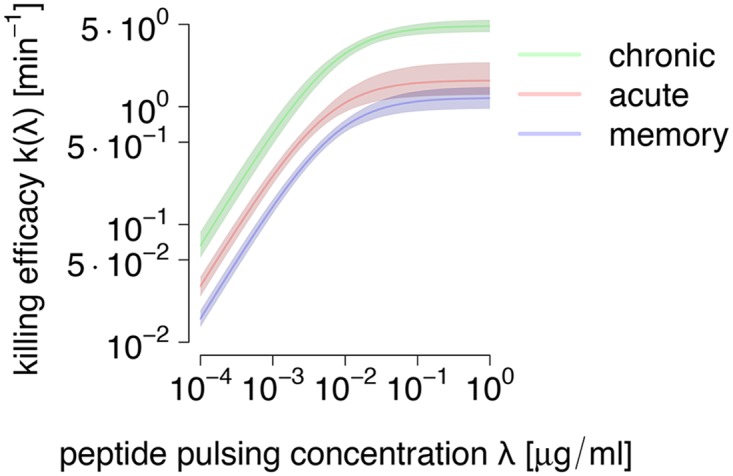
Estimated dependence of killing efficacies on pulsed peptide concentrations *λ* with 95% confidence intervals from bootstrap replicates. The values for the chronic treatment group (green) are clearly larger than values for the acute (red) and memory (blue) groups. The confidence intervals were calculated by determining the 2.5% and 97.5% percentiles of the distribution of $k(k_{\text{max}},\lambda_{\frac{1}{2}},\lambda )$ values for all bootstrapped parameter pairs $\left(k_{\text{max}},\lambda_{\frac{1}{2}}\right)$ (where 1000 parameter pairs were evaluated) at each concentration *λ*.

By using bootstrapping, we were able to calculate 95% confidence bands for the per-cell efficacy profile across all peptide loads (Fig 8.). The CTL’s in the chronically infected mice show larger killing efficacies than the other groups. The acutely infected group reveals significantly larger killing efficacies for concentrations up to $10^{-1}\frac{\mu \text{g}}{\text{ml}}$ compared to mice from the memory group. Thus, although hierachies that change with peptide loads would be theoretically possible in our model, as shown in Fig 5., we infer a clear and unchanging hierarchy across peptide loads.

### (c2) Estimates under saturation of CTL responses

According to the mass-action assumption the killing rate increases linearly with the frequency of CTL. At very high CTL frequencies, however, the killing rate will stop to increase and saturate. Recent studies based on cellular automata predict a threshold in CTL frequencies of 0.03, above which saturation effects in CTLs should not be neglected [25]. Below this threshold, mass action dynamics is an appropriate approximation [26]. In our data, the threshold is exceeded in the acute group (⟨*C*_*a*_⟩ = 0.042) and slightly in the memory group (⟨*C*_*m*_⟩ = 0.031), but not the chronic group (⟨*C*_*p*_⟩ = 0.006).

For this reason, we relaxed the mass-action assumption and assumed that the killing rate saturates with increasing CTL frequencies. Saturation in CTL frequencies implies that the killing efficacy per CTL approaches an upper limit with increasing CTL numbers. The individual CTL killing efficacy is thus impaired by the presence of other CTLs in the same compartment.

If we incorporate the saturation in CTL frequencies into our model for killing, we obtain the following killing rate:

$$\begin{array}{c} f\left(k\left(k_{\max,j},\lambda_{\frac{1}{2},j},\lambda \right),C_{\frac{1}{2},j}\right)=\frac{k_{\max,j}\lambda }{\lambda_{\frac{1}{2},j}+\lambda }\cdot \frac{C_{j}}{C_{\frac{1}{2},j}+C_{j}} \end{array}$$ (5)

Here *j* ∈ {*a*, *p*, *m*} denotes the acute, chronic and memory groups, respectively. $C_{\frac{1}{2},j}$ is the CTL frequency, at which the saturation effects in *C*_*j*_ are half-maximal, and is sometimes confusingly referred to as the *saturation threshold*.

With the adoption of a saturation model, the meaning of the killing efficacy *k*, and with it of the parameter *k*_max_ changes. In the mass action model, *k* is the per cell killing efficacy induced by a peptide load *λ*. Therefore, *k*_max_ signifies the maximum per cell killing efficacy that can be attained by a CTL. However, in the saturation model, *k* and *k*_max_ have a less intuitive meaning. Here, *k* is the actual killing rate exerted by all peptide-specific cells at a particular peptide load *λ* accounting for CTL saturation effects. Hence, in the saturation model, *k*_max_ captures the maximum killing rate that can be reached by the entire CTL population specific to a particular peptide.

While it is conceivable that the half-maximum CTL frequencies $C_{\frac{1}{2},j}$ differ by group, we do not have the resolution in our experimental data to determine these parameters for each group individually. This is due to the very low variation of CTL frequencies in different mice within the groups. We therefore defined killing model variants of increasing complexity, starting with a variant in which all parameters are equal between groups, to variants in which increasing number of parameters differ. We refer to these model variants as Model A, B, C, and D. The choice of these models was biologically motivated, and represents a subset of all theoretically conceivable combinations of inequalities between group parameters.

We performed F-tests between these four models. In model A we assume all maximum killing efficacies within treatment groups to be equal *k*_max,*a*_ = *k*_max,*p*_ = *k*_max,*m*_, all sensitivities on concentration between groups to be equal, $\lambda_{\frac{1}{2},a}=\lambda_{\frac{1}{2},p}=\lambda_{\frac{1}{2},m}$, as well as the saturation levels for CTLs, $C_{\frac{1}{2},a}=C_{\frac{1}{2},p}=C_{\frac{1}{2},m}$. Model B is given by the assumption that sensitivities between the treatment groups are independent or not necessarily equal, $\lambda_{\frac{1}{2},a}\neq \lambda_{\frac{1}{2},p}\neq \lambda_{\frac{1}{2},m}$. Model C is given by assuming an difference between memory cell CTL saturation levels and effector cell saturation levels: $C_{\frac{1}{2},a}=C_{\frac{1}{2},p}\neq C_{\frac{1}{2},m}$. Model D adds further complexity to model C by relaxing the assumption of equal maximum killing efficacies: *k*_max,*a*_ ≠ *k*_max,*p*_ ≠ *k*_max,*m*_.

We found that model B provides a significantly better fit than model A (*p* = 3.53 ⋅ 10^−9^, by F-test). Model C provides a significant improvement of a fit over model B (*p* = 2.21 ⋅ 10^−5^). Intriguingly, model C (six parameters) does also lead to a better fit of the data than the combination of all group-wise fits of the mass-action kinetics model with killing efficacy dependence on pulsing concentrations (also six parameters, identical data). The mass action model leads to a residual sum of squares (RSS) of 3.43, whereas model C leads to an RSS of 3.33. Also when comparing these two models by the Akaike Information Criterion (AIC), model C is associated with a smaller information loss (AIC: 301) than the mass-action model (AIC: 308.14). Here, AIC was calculated as AIC = 2*k* + *n* log(RSS) [27, 28], where the number of parameters in the model is *k* = 6 and the number of observations is *n* = 240, see S1 Text. The fit of model C to the data is shown in S1 Fig. Lastly, model D does not significantly improve the fit compared to model C (*p* = 0.323).

In addition to models A, B and D, we tested several other alternatives to model C, and found that model C is statistically the best supported model (see S1 Text and S2 Fig).

The fact that model C comes out as the best model in our model selection scheme is consistent with biological observations and previous quantitative analyses. Studies which employed mass-action kinetics for low effector-target cell ratios, found no significant differences between the killing efficacy of CTLs in acute and chronic infections [15], as well as between effector and memory CTL [3, 29]. This is in line with the assumption of model C that the maximum killing efficacies of CTLs in all mice groups are equal *k*_max,*a*_ = *k*_max,*p*_ = *k*_max,*m*_. The model assumption of unequal half-maximum CTL frequencies differ between memory and effector CTL is supported by the observation that memory CTL bind to target cells longer until they kill due to lower perforin and granzyme levels [30].

We fitted function (3) under model C to the combined data of all treatment groups. Confidence intervals were obtained by bootstrapping. The estimated values of the model parameters are shown in Table 2. Estimated maximum killing efficacies are smaller compared to the mass-action kinetics estimates, because of a different gauging under the new saturation assumption. The estimates for the peptide load sensitivities are very similar to those in mass-action estimates (shown in Table 1). For the saturation threshold in $C_{\frac{1}{2},\text{effector}}$, we obtain values around 0.015. This estimate is very similar to one obtained by different methods (see S1 Text). The saturation threshold estimate for memory CTL is substantially higher than for CTL in acute and chronic infections.

**Table 2:** Estimates of *k*_max_ and $\lambda_{\frac{1}{2},a}$, $\lambda_{\frac{1}{2},p}$, $\lambda_{\frac{1}{2},m}$, as well as $C_{\frac{1}{2},\text{eff}}$ and $C_{\frac{1}{2},\text{mem}}$ under model C for all treatment groups combined. The confidence intervals were calculated with 1000 bootstrap replicates and the percentile method.

**parameter**	**value**
(a) *k*_max_[min^−1^]	0.095 (0.055, 0.138)
(b) $\lambda_{\frac{1}{2},\text{acute}}[\frac{\mu \text{g}}{\text{ml}}]$	10^−2.23^ (10^−2.44^, 10^−2.10^)
(c) $\lambda_{\frac{1}{2},\text{chronic}}[\frac{\mu \text{g}}{\text{ml}}]$	10^−2.16^ (10^−2.29^, 10^−1.99^)
(d) $\lambda_{\frac{1}{2},\text{memory}}[\frac{\mu \text{g}}{\text{ml}}]$	10^−2.15^ (10^−2.28^, 10^−1.93^)
(e) $C_{\frac{1}{2},\text{effector}}$	0.013 (0.004, 0.023)
(f) $C_{\frac{1}{2},\text{memory}}$	0.051 (0.007, 0.088)

## Discussion

In this study, we have estimated CTL-mediated killing efficacies using *in vivo* killing assays across a wide range of peptide loads. Assuming mass-action kinetics, a clear hierarchy emerged between acute, chronic and memory responses. The chronic group showed a larger killing efficacy per CTL than the acute group, which in turn was more effective than the memory group across the entire range of peptide loads. This clearly shows, consistent with previous studies [15, 20], that CTL during chronic infections are not impaired in their ability to kill. Neither do these cells require more peptide on their targets to kill ($\lambda_{\frac{1}{2}}$), nor do they have a lower killing efficacy for very large peptide loads.

In contrast to our previous work [15], the estimates of the maximal per CTL killing efficacies were around one order of magnitude smaller for the acutely infected mice. This mismatch for the acute group is likely due to CTL frequency saturation effects. Saturation effects are likely to play a smaller role in [15], where due to later cell transfer times (15 days after infection) the average GP33-specific CTL frequency is about 0.01. In this study, the average GP33-specific CTL frequency in acutely infected mice was 0.042 (8 days after infection). As the frequencies of GP33-specific CTL increase, killing efficacies will inevitably enter a saturation regime. In the saturation regime, a further increase in CTL does not lead to more total killing, violating a core assumption of the mass-action model. Erroneously adopting the mass-action assumption under saturation will therefore lead to an underestimate of the per CTL killing efficacy.

In contrast, GP33-specific CTL frequencies for chronically infected mice in the experiments presented in [15] were very similar to those in this study, and no inconsistencies between killing efficacy estimates arise.

To account for possible saturation effects in CTL numbers we also investigated mathematical models accounting for the saturation in killing rates for high CTL levels. Such saturating effects arise when the duration of killing is the limiting factor in the dynamics [31]. By a variety of methods we estimate that GP33-specific CTL need to exceed a frequency of 1% among splenocytes for saturation in acute and chronic infection. This estimate is about two to three times lower than that obtained from cellular automata models [25].

The results for the estimated recognition sensitivities between groups are almost identical between mass-action kinetics and CTL saturation model fits. Irrespective of the mass-action assumption, we did not find significant differences in the recognition sensitivities between responses. These estimates are of the same order of magnitude as comparable estimates found for chronic infections of polyoma virus, but differ for acute infections [16].

This study relies on estimates of the net unpulsed target cell migration into the spleen *μ*(*t*) and target cell data from the blood obtained in previous work [15]. Hence, uncertainties regarding the migration of target cells into the spleen that are associated with the experimental approach will inevitably be inherited by our killing efficacy estimates.

There exist other models for CTL killing, such as the refractory model, epitope decay and CTL exhaustion models [13] or a recently investigated double saturation model [32], that could have been used to fit the data. In this study, we limited the analysis to the standard mass-action kinetics and CTL saturation models. The refractory and exhaustion models were found to contradict biological observations in [13]. The CTL epitope decay model accounts for the observation that peptide-MHC complexes have lifetimes on the order of hours [33], and could be lost over the course of our experimental study. However, in our previous study, this model did not provide significantly better fits to standard models [15]. Furthermore, the model could not provide additional information as to the relative strengths of the responses in the different treatment groups. The double saturation model—which accounts for saturation in both target as well as CTL numbers– could capture the functional response of the killing in a variety of conditions simulated in a computer model of lymphoid tissue. However, the relatively low variance in the target cell numbers in the spleen (0.08−0.12 probability for a target cell to be in the spleen [15]) as well as the low variance of CTL frequencies do not permit to resolve the quantitative details of saturation in our data.

The main motivation for estimating CTL killing efficacies for lower peptide loads in the present study were the discrepancies between killing estimates in vivo across a broad range of study systems [1, 3–16]. However, there are also inconsistencies across in vivo and in vitro studies. It is unresolved if CTL are equally able to kill during chronic and acute LCMV infection. Some studies report cytolytic impairment of CTLs in chronic infections [34–36], while others find unchanged cytotoxic potential in chronic infection [20]. These inconsistencies were thought to arise because the study systems employ either different peptide-MHC densities or different cells [20]. Our study shows that lower peptide-MHC densities by themselves cannot explain the impaired killing ability that some studies found: the killing ability during chronic LCMV infection is at least as strong as during acute infection across all peptide loads we tested in our study.

Our estimates do not completely resolve why there are discrepancies of many orders of magnitude in CTL killing efficacy estimates between HIV, SIV, BLV, HTLV-1 on one hand, and LCMV and polyoma virus on the other. The peptide loads on naturally infected cells correspond to a peptide pulse of approximately $10^{-3}\frac{\mu \text{g}}{\text{ml}}$. This number is estimated from a comparison of the T-cell stimulation by LCMV-infected or peptide-pulsed macrophages. An identical activation level of macrophages is attained for T-cells pulsed at $10^{-9}M\approx 10^{-3}\frac{\mu \text{g}}{\text{ml}}$. The killing efficacies obtained in *in vivo* killing assays with for the commonly applied large pulses of $1\frac{\mu \text{g}}{\text{ml}}$ and the physiologically more realistic pulse of $10^{-3}\frac{\mu \text{g}}{\text{ml}}$ differ by a factor 10. Thus, lower peptide loads explain only up to two orders of magnitude difference in CTL killing rates, and alternative explanations for the remaining large discrepancies need to be sought.

The discrepancies between the estimated killing efficacies could be due to specific differences between the host species and the viruses. For instance, differences in the distribution of MHC molecules on target cells between mice and primates could have important effects. Other important host features, such as size, might affect CTL killing. Virus features are also expected to impact CTL killing efficacy. For example, viral protein expression in infected cells might vary substantially between different viruses and the infected cell type. Additionally, epitopes from different pathogens might elicit differently strong immune responses, which could contribute to CTL killing efficacy variation.

Other studies have previously shown that the peptide concentration employed for target cell pulsing can affect the killing efficacies of CTLs in polyoma virus (PyV) [16, 37]. As in our own study, killing efficacies declined with decreasing pulsing concentrations, indicating that the CTL’s ability to recognize and kill infected target cells depends on the number of peptide/MHC molecules presented on the target cell surface. In contrast to our results, fits of mass-action models do not show a clear hierarchy of killing efficacies between CTLs in acute and chronic infections. Rather, the hierarchy of killing abilities of CTLs in acute and chronic infections is reversed when pulsing concentrations are varied. Although this study differs in scope and in the viral system from the study of [16], it can be regarded as an extension of it. Here, we have additionally investigated saturation models by employing statistical methods established in recent studies (arcsin transformations for adequate error minimization in frequency data [13], estimation of net flux rates of target cells into spleen [15]).

Compared to the primary response after encounter of a pathogen, memory T cells confer a faster and stronger immune response to the host upon re-encounter, granting increased protection [38]. As in previous studies [13], we found that memory CTL do not kill at higher rates than CTL in acute and chronic infections, particularly at lower peptide loads. We therefore have no evidence for an increased sensitivity of memory CTL compared to acute or chronic CTL, as has been suggested [39]. The increased level of viral control that is observed upon re-exposure is better explained by higher initial number or a higher proliferative capacity of memory CTL as compared to naïve CTL.

## Supporting Information

S1 Text Supporting information text. The Supporting Information Text S1 contains an alternative estimation method for the saturation threshold $C_{\frac{1}{2},\text{effector}}$, an explanation of how the model selection of model C was carried out and a mathematical derivation for the formula used for the Akaike Information Criterion calculations in the manuscript.
(PDF)

S1 Fig Fitted curves of Eq (3) assuming saturation in CTL killing (model C) on estimated fractions of killed target cells in all mouse groups. The curves were obtained by using the estimates for the parameters in model C (see Table 2) to plot (3). The panels show the fits to (left) the acute, (middle) the chronic and (right) the memory groups. The dots are the averages of the fraction of killed target cells of all mice in a treatment group.
(TIF)

S2 Fig Selection of model C. Starting from very restrictive assumptions for a model of CTL behavior across treatment groups (top circle), additional relaxing assumptions lead to models with increasing numbers of parameters (circles below, connected by arrows). Whether models derived from more restrictive, nested models provide a significantly better fit to the data is assessed by F-tests. The p-values associated with each relaxed model assumption (arrows) are the outcomes of F-tests between the two models that have been fitted to the data set that includes mice from all treatment groups. The AIC values for each model are given within each model-specifying circle. Model C is selected as the model which increases the quality of the fit most significantly, but does not allow a further relaxation of assumptions.
(TIF)

S1 Data Experimental data. The data used for the analysis is represented as a table. The first column is the name of the sample, the columns denoted with "unpulsed" or "*_gp33" are the relative proportions of the transferred cell subpopulations, pulsed with the corresponding concentrations. "location" denotes whether the data were taken from the blood (1) or from spleen (2). "Time" is the time after transfer at which the samples were taken, in minutes. "mouse.nr" is the number of the mouse examined, where mouse numbers 1-4 belong to the naive, 5-8 to the acute, 9-12 to the chronic and 13-16 to the memory groups. "CD8" is the total frequency and "CD.GP33" is the frequency of GP33-specific CD8+ T cells among splenocytes. The column names "pc*GP33.killed" denote the percentage of cells killed in the subpopulations of the corresponding pulsing concentrations. The rest of the columns were not used for analysis.
(CSV)
