# Supplementary material for: Estimating the In Vivo Killing Efficacy of Cytotoxic T Lymphocytes across Different Peptide-MHC Complex Densities
Source: PLoS Comput Biol. 2015 May 1;11(5):e1004178. doi: 10.1371/journal.pcbi.1004178 (PMC4416789; doi:10.1371/journal.pcbi.1004178)
Supplement: S1 Text — The Supporting Information Text S1 contains an alternative estimation method for the saturation threshold C12,effector, an explanation of how the model selection of model C was carried out and a mathematical derivation for the formula used for the Akaike Information Criterion calculations in the manuscript. (PDF) [file pcbi.1004178.s001.pdf]

Supporting Information

Estimating the in vivo killing efficacy of cytotoxic T lymphocytes across different peptide-MHC complex densities

Victor Garcia[1,*], Kirsten Richter[2], Frederik Graw[3], Annette Oxenius[2], and Roland R. Regoes[1,*]

[1]Institute of Integrative Biology, ETH Zurich, Universitätstr. 16, CH-8092 Zurich, Switzerland

[2]Institute of Microbiology, ETH Zurich, Wolfgang-Pauli-Str. 10, CH-8093 Zurich, Switzerland

[3]Center for Modeling and Simulation in the Biosciences, University of Heidelberg, Im Neuenheimer Feld 267, 69120 Heidelberg, Germany

[*]*corresponding author:* email: victor.garcia@env.ethz.ch, roland.regoes@env.ethz.ch

Estimation of saturation threshold of epitope-specific CTL frequencies

In the total CTL killing rate, the saturation in CTL frequencies is given by:

$$f(k(k_{\max,j}, \lambda_{\frac{1}{2},j}, \lambda), C_{\frac{1}{2},j}) = \frac{k_{\max,j}\lambda}{\lambda_{\frac{1}{2},j} + \lambda} \cdot \frac{C_j}{C_{\frac{1}{2},j} + C_j} \tag{1}$$

Here $j \in \{a, p, m\}$ denotes the acute, persistent (chronic) and memory groups, respectively. $C_{\frac{1}{2},j}$ is the CTL frequency *saturation threshold*. The peptide load on the target cells is λ, and $k_{\max,j}$ is the maximal killing rate, which is half-maximal at $\lambda_{\frac{1}{2},j}$.

For very high pulsing concentrations at the saturation level for the peptide-dependent killing efficacy, the relation (1) can be replaced by:

$$f(k_{\max,j}, C_{\frac{1}{2},j}, C_j) = k_{\max,j} \cdot \frac{C}{C_{\frac{1}{2},j} + C_j}. \tag{2}$$

Here, the total killing rate in a group can be regarded as a given fixed rate f_s, where $s \in \{a, p\}$, for acutely and persistently infected, respectively. If the total killing rate is

fixed and we assume $C_{\frac{1}{2},a} = C_{\frac{1}{2},p} = C_{\frac{1}{2}}$, then $k_{\max,j}$ and $C_{\frac{1}{2}}$ are implicitly linked by (1). Hence,

$$k_{\max,a} = f_a \cdot \frac{C_{\frac{1}{2}} + C_a}{C_a} \tag{3}$$

$$k_{\max,p} = f_p \cdot \frac{C_{\frac{1}{2}} + C_p}{C_p}. \tag{4}$$

These are two linear functions of $k_{\max,a}$ and $k_{\max,p}$ in $C_{\frac{1}{2}}$. The value for $C_{\frac{1}{2}}$, at which they intersect is given by:

$$C_{\frac{1}{2}} = \frac{C_a C_p \left(f_p - f_a\right)}{C_p f_a - C_a f_p}. \tag{5}$$

With estimates of $C_{a,p}$, as well as $f_{a,p}$, it is therefore possible to infer the value of $C_{\frac{1}{2}}$ at which the maximal per CTL killing efficacies of acutely and persistenly infected mice are equal. In order to obtain the confidence intervals, we sample 10^5 times from the confidence distributions around the estimates of $C_{a,p}$ and $f_{a,p}$, taking into account the covariance between them. To obtain a better estimate for this covariance, we assumed the correlation between C_a and f_a to be equal to the correlation between C_p and f_p. We hence measured the covariance between the set $\{C_a, C_p\}$ and the set $\{f_a, f_p\}$. The confidence intervals for $C_{\frac{1}{2}}$ are calculated by the percentile method.

The values for $C_{\frac{1}{2}}$ have been measured directly in the *in vivo* killing assay. We estimated the values of $f_{a,p}$ by setting the epitope-specific CTL levels to one in the killing efficacy dependence model. By that method we obtain more realistic estimates for the total saturation killing rate than by only considering the data at maximum peptide concentrations. This leads to $f_a = 0.062(0.015, 0.109)\ \mathrm{min}^{-1}$, $f_p = 0.031(0.009, 0.052)\ \mathrm{min}^{-1}$. With these estimates and the experimental data for $C_{a,p}$, we obtain that $C_{\frac{1}{2}} = 0.0095$ $(0.0883, -0.010)$. The negative value for the lower bound of the CI stems from the fact that some of the samples combinations do only have negative solutions for $C_{\frac{1}{2}}$. This is true for 13.5% of cases.

Model selection of model C

To gain more confidence in model C, we performed further F-tests on alternatives to model C that are derived from model B and have equal numbers of parameters. There exist five alternatives to model C with an equal number of independent parameters. Four ways exist to further relax assumptions in model C by adding one single parameter. These alternative models also allow unequal values of $k_{\max}$ between treatments groups.

Of all five ways to further relax the assumptions made in model B by adding one more parameter, model C provides by far the best improvement, with a p-value of about two orders of magnitude below the next best alternative model. Note that this

also includes models where the equality between killing efficacies in different models is relaxed. Relaxing the assumptions further to include one more parameter than in model C, does not significantly improve fits (see S1 Fig.).

Calculation of Akaike Information criterion measures

In this section we show how the Akaike Information Criterion (AIC) values were calculated in the manuscript and in S2 Fig. The AIC is a measure employed for the assessment of the information loss associated with a model fit to data. The larger the AIC, the higher the information loss and the less desirable the model in relation to other models. We begin with the definition of the AIC:

$$\mathrm{AIC} = 2k - 2l(\Theta), \tag{6}$$

where k is the number of free parameters in the model, and $l(\Theta)$ is the maximized value of the log-likelihood of the model fitted to the data. In the text below, we are going to follow the exposition by Mohanan [27] as well as Burnham and Anderson [28] to clarify how we calculated the AIC.

The assessment of the number of parameters is straight forward in the models explored in the manuscript. To calculate the maximum log-likelihood, we use its relationship to the sum of squared residuals (SSR), which is the output of the optimization procedures utilized to fit the models to the data.

In non-linear regression, the observations y_i, where $i \in \{1, \ldots, n\}$ are being used to fit a non-linear model $g(\mathbf{x}_i, \Theta)$, where $\mathbf{x}_i$ is the the vector of predictors for the ith observation, and Θ is a k-dimensional vector of parameters. Since a value exists for each i of the model function g, we can the define a vector $\mathbf{g}(\Theta)$, where the ith entry corresponds to $g(\mathbf{x}_i, \Theta)$. In non-linear regression, it is commonly assumed that:

$$\mathbf{Y} \sim N_n(\mathbf{g}(\Theta), \sigma^2 \mathbf{I}_n), \tag{7}$$

where σ^2 is the variance of the errors and $\mathbf{I}_n$ is the unity matrix of dimension n. From this relation, it follows that the corresponding distribution of observations should be:

$$L(\Theta, \sigma^2) = (2\pi\sigma^2)^{-\frac{n}{2}} \exp(-\sum_i^n \frac{(y_i - g(\mathbf{x}_i, \Theta))^2}{2\sigma^2}). \tag{8}$$

Interpreted as a function of Θ, this is the likelihood function of the non-linear model. Using the the following notation for the sum of squared residuals (SSR):

$$S(\Theta) = \sum_i^n (y_i - g_i(\mathbf{x}_i, \Theta))^2, \tag{9}$$

we can proceed to calculate the log-likelihood:

$$l_n(\Theta, \sigma^2) = \log(L(\Theta, \sigma^2)) = -\frac{n}{2}\log(2\pi) - \frac{n}{2}\log(\sigma^2) - \frac{S(\Theta)}{2\sigma^2}. \tag{10}$$

Since the maximum likelihood estimator of σ^2 is proportional to the SSR, that is,

$$\hat{\sigma}^2 = S(\Theta)/n, \tag{11}$$

we find that the log likelihood is directly proportional to the logarithm of the SSR, and that maximizing the likelihood is equivalent to minimizing the SSR:

$$l_n(\Theta) = \text{constant} - (n/2)\log(S(\Theta)). \tag{12}$$

In most applications involving AIC values, the constant terms which only depend on the number of observations are neglected, since AIC values do only confer meaning when compared to AIC values of other models applied to the same data set.

The non-constant terms are identical to those implemented in *logLik* [23], an R-based function to calculate the log-likelihood from, for instance, nonlinear-regression estimates of the SSR found by the R-function *nls* [23].

In the manuscript, we therefore calculated the AIC values in the following way:

$$\text{AIC} = 2k + n\log(S(\Theta)). \tag{13}$$

In the study presented here, we have $n = n_g \cdot n_{\text{gs}} \cdot n_t \cdot n_p = 240$, where $n_g = 3$ is the number of mice groups utilized for model fitting, $n_{\text{gs}} = 4$ is the number of mice per group, n_t the number of measured time points and $n_p = 5$ is the number of different peptide loads employed.

References

[23] R Development Core Team. R: A Language and Environment for Statistical Computing. Vienna, Austria; 2012. ISBN 3-900051-07-0. Available from: `http://www.R-project.org/`.

[27] Monahan JF. Numerical methods of statistics. Cambridge University Press; 2001.

[28] Burnham KP, Anderson DR. Model selection and multimodel inference: a practical information-theoretic approach. Springer; 2002.
