# Supplementary figures and images for: Estimating the In Vivo Killing Efficacy of Cytotoxic T Lymphocytes across Different Peptide-MHC Complex Densities
Source: PLoS Comput Biol. 2015 May 1;11(5):e1004178. doi: 10.1371/journal.pcbi.1004178 (PMC4416789; doi:10.1371/journal.pcbi.1004178)

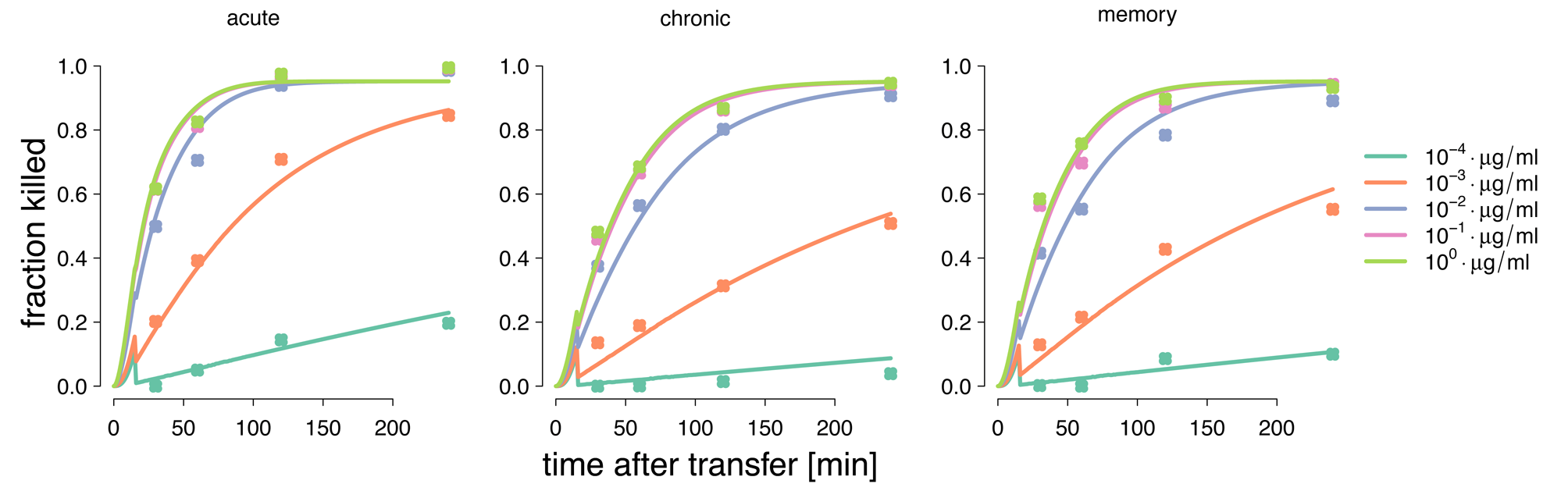

Supplement: S1 Fig — The curves were obtained by using the estimates for the parameters in model C (see Table 2) to plot (3). The panels show the fits to (left) the acute, (middle) the chronic and (right) the memory groups. The dots are the averages of the fraction of killed target cells of all mice in a treatment group. (TIF) [file pcbi.1004178.s002.tif]

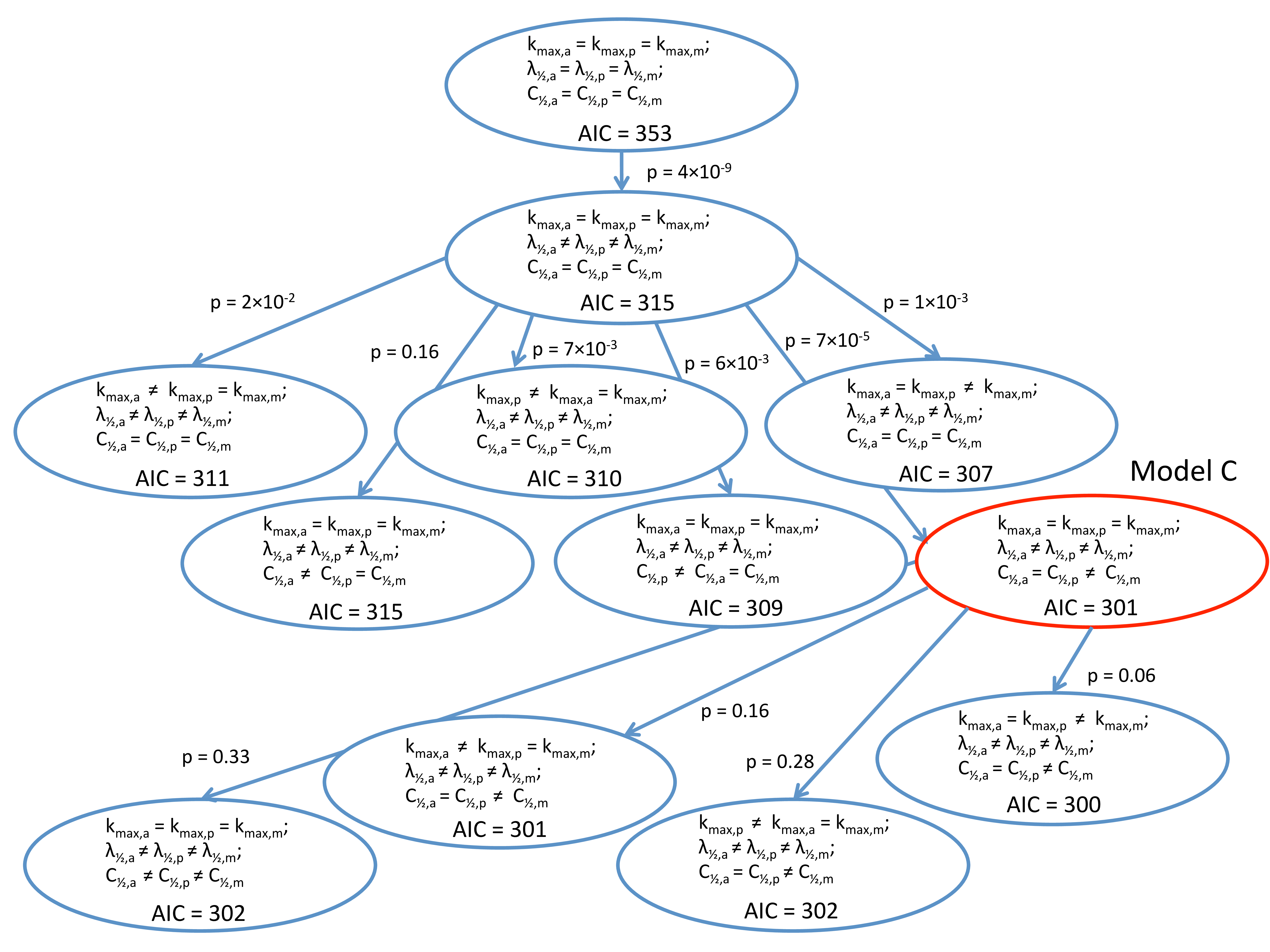

Supplement: S2 Fig — Starting from very restrictive assumptions for a model of CTL behavior across treatment groups (top circle), additional relaxing assumptions lead to models with increasing numbers of parameters (circles below, connected by arrows). Whether models derived from more restrictive, nested models provide a significantly better fit to the data is assessed by F-tests. The p-values associated with each relaxed model assumption (arrows) are the outcomes of F-tests between the two models that have been fitted to the data set that includes mice from all treatment groups. The AIC values for each model are given within each model-specifying circle. Model C is selected as the model which increases the quality of the fit most significantly, but does not allow a further relaxation of assumptions. (TIF) [file pcbi.1004178.s003.tif]
